# Ectopic PTH-producing parathyroid cyst inside the thymus: a case report

**DOI:** 10.1186/s12902-022-01256-4

**Published:** 2022-12-21

**Authors:** Haruka Takenouchi, Takatoshi Anno, Ayaka Harada, Hayato Isobe, Yukiko Kimura, Fumiko Kawasaki, Kohei Kaku, Koichi Tomoda, Hideyo Fujiwara, Hideaki Kaneto

**Affiliations:** 1 Department of General Internal Medicine 1, Kawasaki Medical School, 2-6-1 Nakasange, Kita-ku, Okayama, 700-8505 Japan; 2 Department of Pathology, Kawasaki Medical School, Okayama, 700-8505 Japan; 3 Department of Diabetes, Metabolism and Endocrinology, Kawasaki Medical School, Kurashiki, 701-0192 Japan

**Keywords:** PTH, Ectopic parathyroid glands, Hypercalcemia, Hyperparathyroidism, Mediastinal tumor

## Abstract

**Background:**

The hallmark of hyperparathyroidism is hypersecretion of parathyroid hormone (PTH) which results in hypercalcemia and hypophosphatemia. While hypercalcemia due to malignancy is often brought about by PTH-related protein in adults, PTH-producing tumors are quite rare in clinical practice. Additionally, from the point of embryology, it is very difficult to examine ectopic PTH-producing tissue such as ectopic parathyroid glands. Furthermore, clear histopathological criteria are not present.

**Case presentation:**

A 57-year-old woman was referred to our hospital for hypercalcemia. Her parathyroid hormone (PTH) level was elevated, but there were no enlarged parathyroid glands. Although 99mTc-MIBI confirmed a localized and slightly hyperfunctioning parathyroid tissue in the anterior mediastinum, it was not typical as hyperfunctioning parathyroid. We finally diagnosed her as ectopic PTH-producing cyst-like tumor with venous sampling of PTH. She underwent anterosuperior mediastinal ectopic PTH-producing cyst-like tumor resection. It is noted that intact-PTH concentration of the fluid in the cyst was very high (19,960,000 pg/mL). Based on histopathological findings, we finally diagnosed her as ectopic PTH-producing parathyroid cyst inside the thymus. After resection of anterosuperior mediastinal thymus including ectopic PTH-producing parathyroid cyst, calcium and intact-PTH levels were decreased, and this patient was discharged without any sequelae.

**Conclusions:**

We should know the possibility of superior mediastinal ectopic PTH-producing parathyroid cyst inside the thymus among subjects with ectopic PTH-producing parathyroid glands. Particularly when the cyst is present in the superior mediastinum, it is necessary to do careful diagnosis based on not only positive but also negative findings in 99mTc-MIBI. It is noted that the patient’s bloody fluid in the cyst contained 19,960,000 pg/mL of intact-PTH, and its overflow into blood stream resulted in hyperparathyroidism and hypercalcemia. Moreover, in such cases, the diagnosis is usually confirmed after through histological examination of ectopic PTH-producing parathyroid glands. We think that it is very meaningful to let clinicians know this case.

**Supplementary Information:**

The online version contains supplementary material available at 10.1186/s12902-022-01256-4.

## Background

Hallmark of hyperparathyroidism is hypersecretion of parathyroid hormone (PTH) which results in hypercalcemia and hypophosphatemia [1, 2]. PTH is produced in the parathyroid gland after birth by replacing PTH-related protein (PTHrP). PTH and PTHrP function similarly at a fetal stage. While hypercalcemia due to malignancy is often brought about by PTHrP production in adults [3, 4], PTH-producing tumors are quite rare in clinical practice. Indeed, there are only a few cases with convincingly documentation due to the difficulty in precise diagnosis of such tumors [5]. Additionally, from the aspect of embryology, it is quite difficult to examine ectopic PTH-producing tissue such as ectopic parathyroid glands. Furthermore, clear histopathological criteria are not present.

Here, we report a case of ectopic PTH-producing parathyroid cyst inside the thymus. It is noted that the patient’s bloody fluid in the cyst contained 19,960,000 pg/mL of intact-PTH, and its overflow into blood stream resulted in hyperparathyroidism and hypercalcemia. We think that it is very meaningful to let clinicians know this case.

## Case presentation

A 57-year-old woman was referred to our hospital for recent diagnosis of hypercalcemia. Her history included hypertension at age 56 and she was taking no medication. She had no family history. On physical examination, she had symptoms of appetite loss and 4 kg of decreased body weight for a few months. Her height and body weight were 158.9 cm and 48.5 kg. Her vital signs were: temperature, 36.5 °C; blood pressure, 110/72 mmHg; heart rate, 110 beats/min; oxygen saturation, 98% (room air). Laboratory data were as follows: white blood cell count, 5620 /μL (neutrophil 74.1%); red blood cell count, 504 × 10^4^ /μL; hemoglobin, 15.4 g/dL; platelet, 17.8 × 10^4^ /μL. Liver function was within normal range, but she had slight renal dysfunction (creatinine (CRE), 0.89 mg/dL; blood urea nitrogen (BUN) 15 mg/dL, aspartate aminotransferase (AST), 22 U/L; alanine transaminase (ALT), 12 U/L; alkaline phosphatase (ALP), 79 U/L; γ-glutamyl transpeptidase (γ-GTP), 25 U/L; lactate dehydrogenase (LDH), 178 U/L). Her electrolyte data showed elevated calcium and decreased phosphates as follows: sodium, 140 mmol/L; potassium 3.5 mmol/L; chloride 103 mmol/L; inorganic phosphorus 2.0 mg/dL; calcium 14.7 mg/dL; magnesium 1.8 mg/dL. In addition, she had elevated PTH level: intact-PTH, 547 pg/mL; PTHrP, < 1.0 pmol/L; 1.25(OH)2 vitamin D, 71.9 pg/mL; 25-dihydroxy vitamin D, 10.2 ng/mL.

**Fig. 1:**
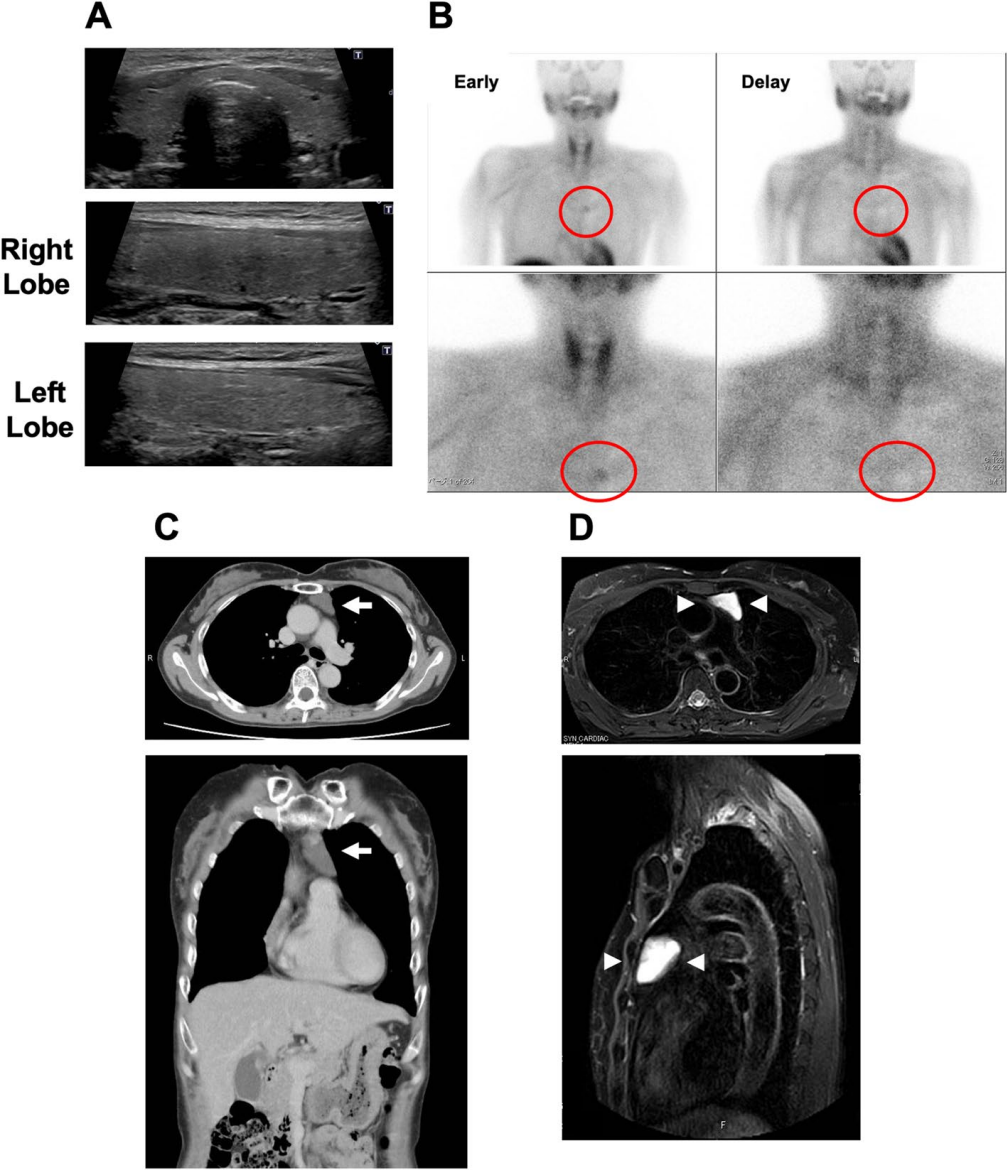
Local diagnosis of ectopic parathyroid cyst inside the thymus in ultrasound examination. (**A**) ^99m^Tc-methoxy-isobutyl-isonitrile scintigraphy (99mTc-MIBI) (**B**), enhanced computed tomography (CT) (**C**) and magnetic resonance imaging (MRI) (D). 99mTc-MIBI confirmed a localized and slightly hyperfunctioning parathyroid tissue in the anterior mediastinum only in an early phase, but not in a delay phase (red circle). Enhanced CT showed a cyst-like tumor in the upper anterior mediastinum (white arrow). The diameter was as large as 50 mm. MRI showed high signal on T1-weighted images due to fluid cyst contents (white arrow head) as observed in 99mTc-MIBI

Ultrasound examination of the parathyroid did not detect enlarged parathyroid gland (Fig. 1A). We performed 99mTc-methoxy-isobutyl-isonitrile scintigraphy (99mTc-MIBI), suspecting the possibility of ectopic parathyroid glands. 99mTc-MIBI confirmed a localized and slightly hyperfunctioning parathyroid tissue in the anterior mediastinum in only early phase, which was not typical of hyperfunctioning parathyroid because of negative spot in delay phase (Fig. 1B). On the other hand, neck and chest computed tomography (CT) and enhanced CT showed a cyst-like tumor in the upper anterior mediastinum, as observed in 99mTc-MIBI (Fig. 1C). The diameter of the tumor was as large as 50 mm. Magnetic resonance imaging (MRI) showed high signal on T1-weighted images due to fluid cyst contents (Fig. 1D) and somatostatin receptor scintigraphy did not detect any positive spots (data not shown). Since we suspected ectopic PTH-producing cyst-like tumor in the upper superior mediastinum, we performed venous sampling of PTH (Supplementary Fig. 1). Intact-PTH levels showed a step up in the territory of the central left branchiocephalic vein compared with both right and left subclavian vein and right atrium (branchiocephalic vein, 1205 pg/mL; right subclavian vein, 256 pg/mL; left subclavian vein, 259 pg/mL; right atrium, 252 pg/mL), corresponding to the anatomic localization of the cyst-like tumor in the previous imaging studies. Based upon these findings, we finally diagnosed her as ectopic PTH-producing cyst-like tumor.

**Fig. 2:**
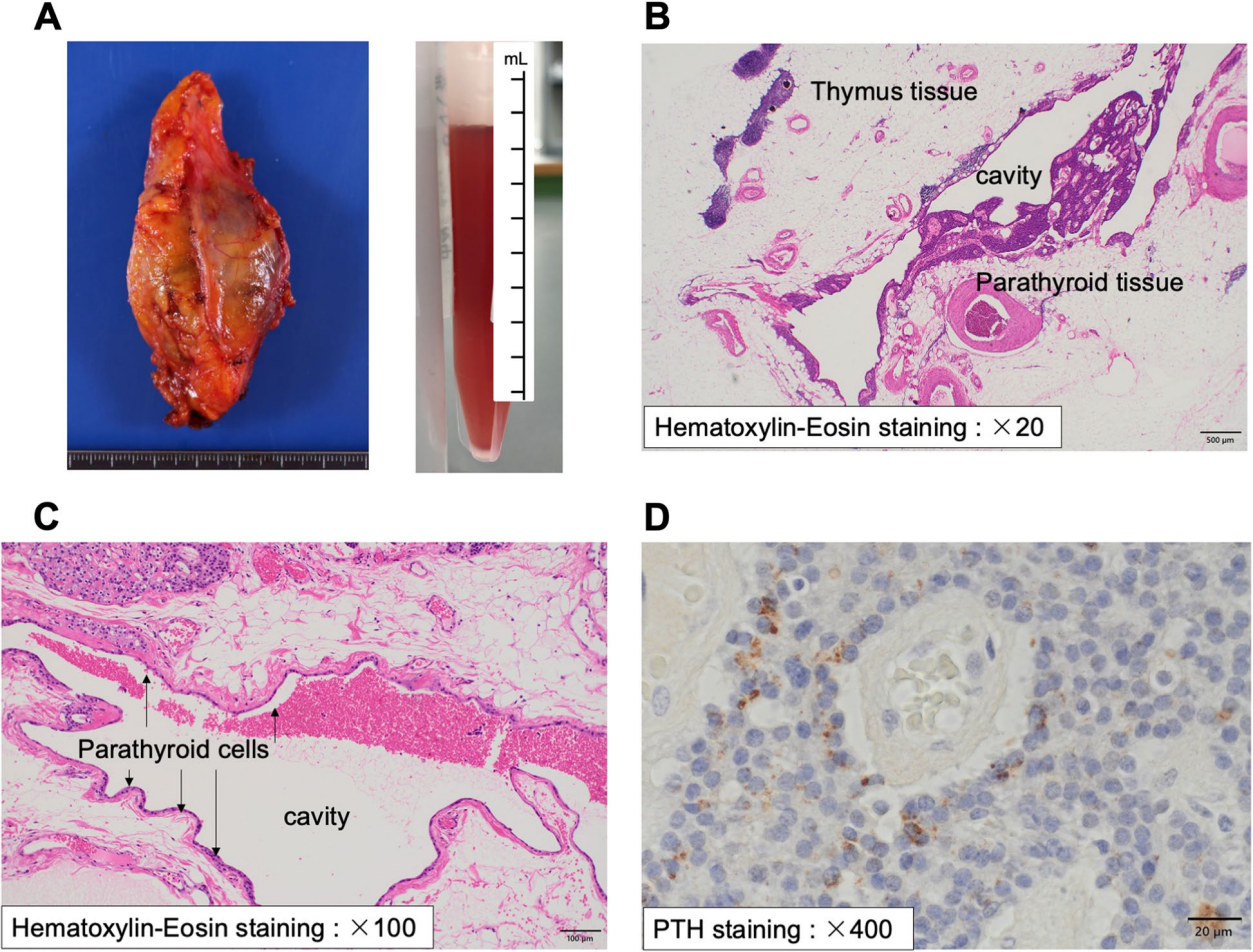
Pathohistological diagnosis of ectopic parathyroid cyst inside the thymus. In histopathological macroscopic findings after thymectomy, bloody fluid was observed in cyst containing 19,960,000 pg/mL of intact-PTH (**A**). In histopathological microscopic findings with Hematoxylin-Eosin staining (B (× 20) and C (× 100)), the formation of cavity of cyst was surrounded by parathyroid cells (hyperplastic glands (**B**) or lining parathyroid cells (**C**)) which was inside the thymus and fatty tissue. Immunohistochemical staining of PTH (D (× 400)) was positive

**Fig. 3:**
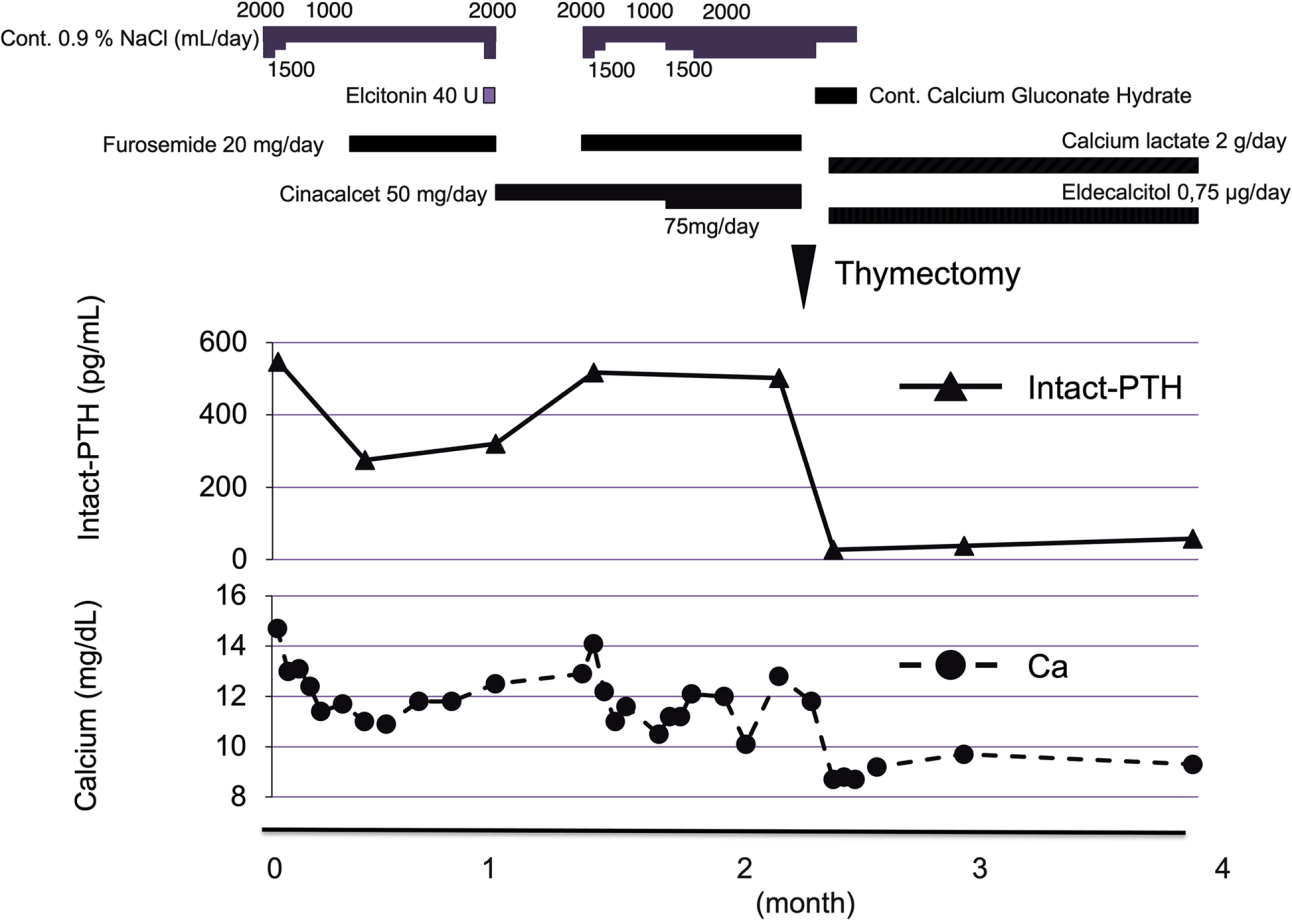
Time course of clinical parameters in this subject. After thymectomy, this patient was discharged without any complications

She underwent anterosuperior mediastinal ectopic PTH-producing cyst-like tumor resection (suspect of thymic cyst and/or adenoma, parathyroid cyst and/or adenoma). It is noted that intact-PTH concentration in the fluid in the cyst was very high (19,960,000 pg/mL) (Fig. 2A). As shown Fig. 2B and C, the formation of cavity of the cyst was surrounded by parathyroid cell inside the thymus and fatty tissue in histopathological microscopic findings. In histopathological microscopic findings, there was no evidence of malignancy in the thymus and parathyroid tissue. Immunohistochemical staining of parathyroid tissue was positive for chromogranin A (weakly), cytokeratin AE1/AE3 (diffuse), Ki-67 (weakly), and negative for MIC-2. In addition, immunohistochemical staining was positive for PTH (Fig. 2D). Based upon these findings, we finally diagnosed her as ectopic PTH-producing parathyroid cyst inside the thymus. After resection of anterosuperior mediastinal thymus including ectopic PTH-producing parathyroid cyst, this patient was discharged without any sequelae, and calcium level was 8.7 mg/dL and intact-PTH level was 27 pg/mL (Fig. 3).

## Discussion and conclusions

Here, we report a case with elevation of serum PTH concentration, but not PTHrP, resulting in hypercalcemia and hyperparathyroidism, which was caused by ectopic PTH-producing parathyroid cyst inside the thymus. It is very difficult to examine ectopic PTH-producing tissue, and there are no clear histopathological criteria. Therefore, understanding the normal process of parathyroid development might provide useful information about how and why ectopic parathyroid adenomas and/or cysts are formed. Generally, the parathyroid glands develop in tandem with the thymus in the third pharyngeal pouch and with the ultimobranchial bodies in the fourth pharyngeal pouch. After then, the combined parathyroid-thymus primordial migrates posteriorly and/or ventrally and separates from each other. After the separation, the parathyroids finish migrating and form two sets of bilateral parathyroid glands, while the thymic lobes migrate to the anterior mediastinum [6–9]. Therefore, it is likely that most ectopic parathyroid adenomas and/or cysts are due to failure at an embryologic stage, and located around the neck [10, 11]. Since ectopic parathyroid gland is rare disease, local and pathohistological diagnosis is very important for a precise diagnosis of ectopic hyperparathyroidism.

When we examine patients with hyperparathyroidism, most cases are primary hyperparathyroidism, which is currently the most common cause of hypercalcemia. Therefore, we usually perform imaging examinations, such as ultrasonography, CT, or MRI of the neck. In imaging tests, approximately 95% of primary hyperparathyroidism is observed as a simple sporadic parathyroid gland [12]. Ultrasonography and CT are quite effective to distinguish a solid mass from a cystic mass around the neck [13]. Since mitochondria-rich oxyphil cells can uptake 99mTc-MIBI in parathyroid lesions [14], 99mTc-MIBI can be quite useful to locate PTH-producing parathyroid gland.

Ectopic parathyroid glands are any parathyroid glands which are not just around the thyroid gland and account for only 15% of all parathyroid adenomas. Moreover, a few percentages of them are present in mediastinal or substernal region, and about 80% of mediastinal cases of ectopic parathyroid glands are around the thymus [15, 16]. In subjects with ectopic parathyroid glands, 99mTc-MIBI is quite helpful, and it is reported the detection rate of ectopic parathyroid gland in 99mTc-MIBI is approximately 85% [17]. However, it is often difficult to detect non-functional ectopic parathyroid glands as a positive spot [18, 19].

Clear criteria to diagnose ectopic parathyroid adenoma or cyst are not present. Parathyroid cysts, which account for less than 1% of cystic neck mass [20], are also rare benign tumors in the head and neck. Location of parathyroid cysts is quite wide from the angle of the mandible to the mediastinum (the left thyroid lobe, 32%; the superior mediastinum,19%; the cervical location, 13%) [21]. In addition, parathyroid cysts are often misdiagnosed as thyroid cysts as well as thyroglossal duct cysts, brachial cleft cysts, thyroid adenomas, and parathyroid carcinoma [21]. Ninety % of parathyroid cysts are non-functioning, and PTH-producing parathyroid cyst is rare [22]. CT is quite useful to distinguish a solid mass or a cystic mass. Parathyroid cyst is often observed as a cyst with a uniform low absorptive area in CT, and peripheral contrast effects are observed in enhanced CT [23]. In subjects with functional parathyroid cyst, MRI also may show high signal on T1-weighted images due to bloody cyst contents. Since it can be difficult to perform differential diagnosis of parathyroid cysts, especially ectopic cysts, from other cysts around the neck, fine needle aspiration (FNA) of cyst is quite effective, and the presence of PTH in the aspirate can lead to the precise diagnosis of parathyroid cyst [24, 25].

In our case, we performed various imaging examinations for hypercalcemia and hyperparathyroidism. First, the lesion was not detected as a simple sporadic parathyroid gland in ultrasonography and CT of the neck, although a cystic mass with a uniform low absorptive area was detected and peripheral contrast effects were observed in CT and enhanced CT. Therefore, we started screening ectopic parathyroid glands and suspected with ectopic superior mediastinal parathyroid cyst. 99mTc-MIBI confirmed a localized and slightly hyperfunctioning parathyroid tissue in the superior mediastinum only in an early phase which is not typical, although 99mTc-MIBI is often useful for functional ectopic parathyroid glands. MRI showed high signal on T1-weighted images, and we diagnosed fluid cyst in enhanced CT and MRI. We had a little evidence to make a clear diagnosis for ectopic functional parathyroid cyst. Somatostatin receptor scintigraphy was negative and the location of superior mediastinal cyst was difficult in FNA. Since 90% of parathyroid cysts are non-functioning and 99mTc-MIBI was not typical as hyperfunctioning parathyroid in this case, we could not deny that this fluid cyst did not contain PTH. Therefore, we performed venous sampling of PTH and found that superior mediastinal cyst was functional PTH-producing cyst. Since the patient’s serum calcium and intact-PTH level decreased to within the normal range after removal of the thymus including ectopic parathyroid cyst, suggesting that diagnosis was appropriately performed.

Since pathohistological diagnosis of ectopic PTH-producing tumors is very difficult, there are few cases in which the presence of PTH secretion from thymoma is shown [26]. Classification of ectopic parathyroid cyst is based on the presence of functionality and symptoms due to hyperparathyroidism and hypercalcemia [27]. Therefore, it is very difficult to know which cells produce PTH such as ectopic parathyroid gland or tumor tissue. While it is thought that non-functioning parathyroid cysts are true simple cysts with an epithelial lining and are more frequently observed compared to functioning cysts, histologic degeneration of an adenoma or hyperplastic gland is observed in functional parathyroid cysts [28, 29]. Since histopathologic examination demonstrates smooth cystic lesions lined with cuboidal epithelium and parathyroid cells in many cases [29], the diagnose should be based on the histopathological appearance of the cyst along with calcium and PTH levels in the fluid obtained with FNA or post-surgical excision [30].

In this case, intact-PTH concentration of the fluid inside the cyst was very high (19,960,000 pg/mL), which led to hyperparathyroidism and hypercalcemia. Most important point in this case was histopathological diagnosis of PTH-producing cell. Immunohistochemical staining of this tissue was not positive for cytokeratin AE1/AE3 and MIC-2, which indicated that this tumor was not thymoma and there was no evidence of malignancy. In addition, the formation of cavity of cyst was surrounded by parathyroid cell which was inside the thymus and fatty tissue clearly showed that this tumor was superior mediastinal ectopic PTH-producing parathyroid cyst inside the thymus. In this case, the patient suffered from hyperparathyroidism and hypercalcemia in adulthood. These results suggest that stored PTH in the cyst overflew into blood stream, leading to hyperparathyroidism and hypercalcemia.

We should bear in mind the possibility of superior mediastinal ectopic PTH-producing parathyroid cyst inside the thymus among subjects with ectopic PTH-producing parathyroid glands. Particularly when the cyst is present in the superior mediastinum, it is necessary to do carefully diagnosis based on not only positive but also negative findings in 99mTc-MIBI. Moreover, in such cases, the diagnosis is usually confirmed after through histological examination of ectopic PTH-producing parathyroid glands.

## Supplementary Information

**Additional file 1: Supplementary Fig. 1.** Venous sampling of PTH. Intact-PTH levels showed a step up in the territory of the central left branchiocephalic vein compared with both right and left subclavian vein and right atrium (branchiocephalic vein, 1205 pg/mL; right subclavian vein, 256 pg/mL; left subclavian vein, 259 pg/mL; right atrium, 252 pg/mL), corresponding to the anatomic localization of the cystic-like tumor in previous imaging studies.

## Data Availability

Not applicable.

## Abbreviations

PTH: Parathyroid hormone
PTHrP: PTH-related protein
CRE: Creatinine
BUN: Blood urea nitrogen
AST: Aspartate aminotransferase
ALT: Alanine transaminase
ALP: Alkaline phosphatase
γ-GTP: γ-glutamyl transpeptidase
LDH: Lactate dehydrogenase
99mTc-MIBI: 99mTc-methoxy-isobutyl-isonitrile scintigraphy
CT: Computed tomography
MRI: Magnetic resonance imaging
FNA: Fine needle aspiration
