# Supplementary figures and images for: Ectopic PTH-producing parathyroid cyst inside the thymus: a case report
Source: BMC Endocr Disord. 2022 Dec 21;22:327. doi: 10.1186/s12902-022-01256-4 (PMC9769032; doi:10.1186/s12902-022-01256-4)

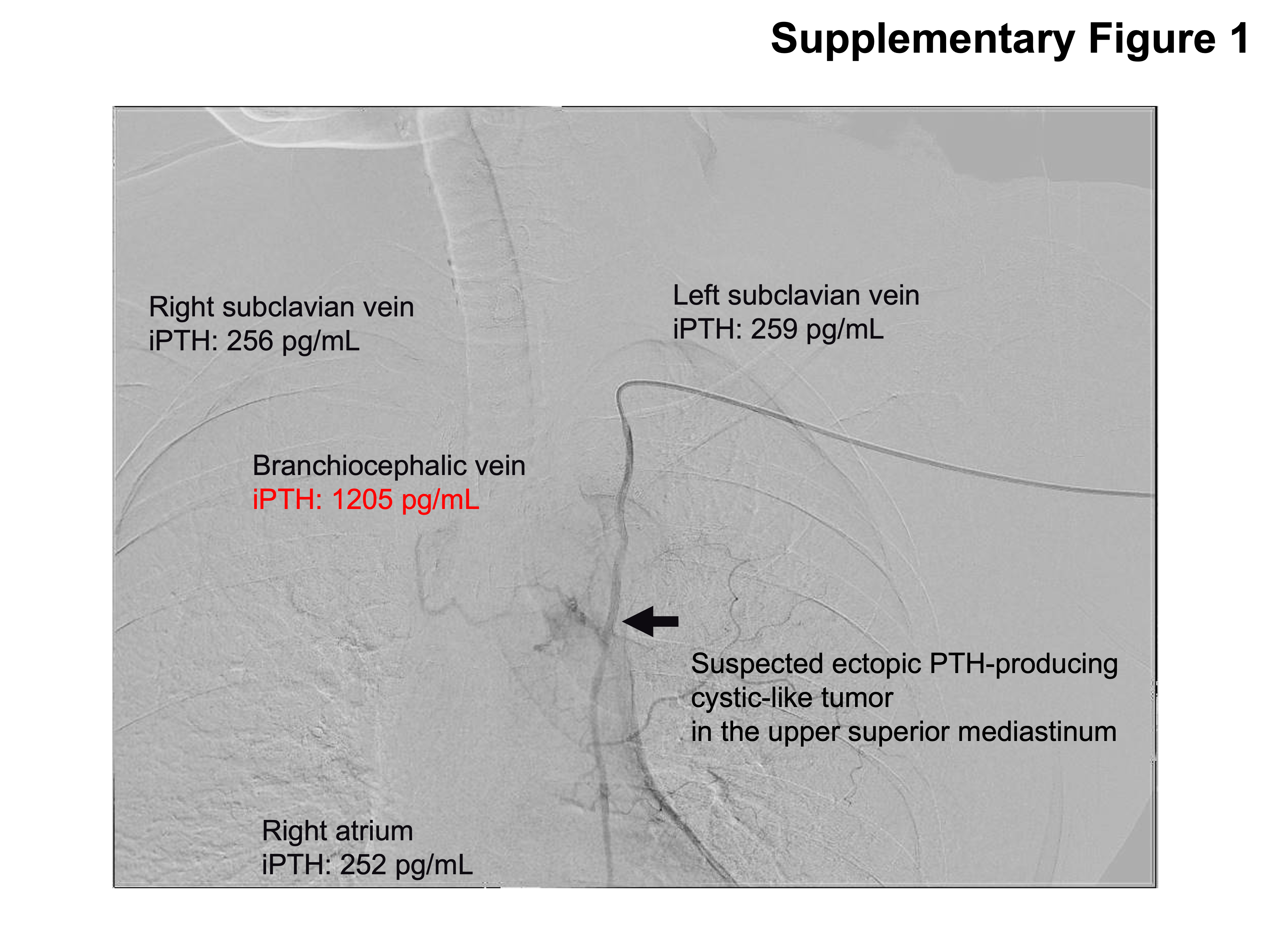

Supplement: Supplementary file 1 — Additional file 1: Supplementary Fig. 1. Venous sampling of PTH. Intact-PTH levels showed a step up in the territory of the central left branchiocephalic vein compared with both right and left subclavian vein and right atrium (branchiocephalic vein, 1205 pg/mL; right subclavian vein, 256 pg/mL; left subclavian vein, 259 pg/mL; right atrium, 252 pg/mL), corresponding to the anatomic localization of the cystic-like tumor in previous imaging studies. [file 12902_2022_1256_MOESM1_ESM.tiff]
